# High levels of viral load monitoring and viral suppression under Treat All in Rwanda – a cross‐sectional study

**DOI:** 10.1002/jia2.25543

**Published:** 2020-06-14

**Authors:** Jonathan Ross, Muhayimpundu Ribakare, Eric Remera, Gad Murenzi, Athanase Munyaneza, Donald R Hoover, Qiuhu Shi, Sabin Nsanzimana, Marcel Yotebieng, Denis Nash, Kathryn Anastos, Central Africa IeDEA

**Affiliations:** ^1^ Division of General Internal Medicine Montefiore Medical Center/Albert Einstein College of Medicine Bronx NY USA; ^2^ Institute of HIV/AIDS Disease Prevention and Control Rwanda Biomedical Center Kigali Rwanda; ^3^ Research Division Rwanda Military Hospital Kigali Rwanda; ^4^ Department of Statistics and Biostatistics and Institute for Health Health Care Policy and Aging Research Rutgers the State University of New Jersey NJ USA; ^5^ Department of Epidemiology and Community Health New York Medical College Valhalla NY USA; ^6^ Institute for Implementation Science in Population Health City University of New York New York NY USA

**Keywords:** Treat All, ARV, HIV care continuum, LMIC, viral load monitoring, viral suppression

## Abstract

**Introduction:**

Aiming to reach UNAIDS 90‐90‐90 targets, nearly all sub‐Saharan African countries have expanded antiretroviral therapy (ART) to all people living with HIV (PLWH) (Treat All). Few published data exist on viral load testing and viral suppression under Treat All in this region. We assessed proportions of patients with available viral load test results and who were virally suppressed, as well as factors associated with viral suppression, among PLWH in 10 Rwandan health centres after Treat All implementation.

**Methods:**

Cross‐sectional study during 2018 of adults (≥15 years) engaged in HIV care at 10 Rwandan health centres. Outcomes were being on ART (available ART initiation date in the study database, with no ART discontinuation prior to 1 January 2018), retained on ART (≥2 post‐ART health centre visits ≥90 days apart during 2018), available viral load test results (viral load measured in 2018 and available in study database) and virally suppressed (most recent 2018 viral load <200 copies/mL). We used modified Poisson regression models accounting for clustering by health centre to determine factors associated with being virally suppressed.

**Results:**

Of 12,238 patients, 7050 (58%) were female and 1028 (8%) were aged 15 to 24 years. Nearly all patients (11,933; 97%) were on ART, of whom 11,198 (94%) were retained on ART. Among patients retained on ART, 10,200 (91%) had available viral load results; of these 9331 (91%) were virally suppressed. Viral suppression was less likely among patients aged 15 to 24 compared to >49 years (adjusted prevalence ratio (aPR): 0.83, 95% CI 0.76 to 0.90 and those with pre‐ART CD4 counts of <200 compared to ≥500 cells/mm^3^ (aPR: 0.92, 95% CI 0.90 to 0.93). There was no statistically significant difference in viral suppression among patients who entered after Treat All implementation compared to those who enrolled before 2010 (aPR 0.98, 95% CI 0.94 to 1.03).

**Conclusions:**

In this large cohort of Rwandan PLWH receiving HIV care after Treat All implementation, patients in study health centres have surpassed the third UNAIDS 90‐90‐90 target. To ensure all PLWH fully benefit from ART, additional efforts should focus on improving ART adherence among younger persons.

## 1 INTRODUCTION

With the goal of ending the global AIDS epidemic, the Joint United Nations Programme on HIV/AIDS (UNAIDS) set the 90‐90‐90 targets with the aim that by 2020, 90% of all people living with HIV (PLWH) know their HIV status, 90% of PLWH with diagnosed HIV infection will receive sustained ART and 90% of all people receiving ART achieve viral suppression [1]. To reach these targets, nearly all countries in sub‐Saharan Africa (SSA) have adopted the World Health Organization (WHO) 2015 recommendation to provide antiretroviral therapy (ART) to all PLWH regardless of clinical stage or CD4 count (“Treat All”) [2]. Most PLWH in SSA are now initiating ART soon after diagnosis [3], with potential to reduce individual‐ and population‐wide morbidity and mortality and decrease onward transmission of HIV.

Viral load monitoring is essential to ensure appropriate clinical decision making for PLWH, identify groups at risk of poor clinical outcomes and determine progress towards the 90‐90‐90 goals. Accordingly, the WHO recommends routine viral load testing at six months after ART initiation and at least annually thereafter [4]. Reduced viral load cost and resource re‐allocation (i.e. reducing use of CD4 test and scaling up use of viral load) have made viral load testing more accessible [5]. However, availability of viral load testing in SSA is highly variable, with recent analyses demonstrating that in many settings, fewer than half of PLWH on ART received a routine viral load test per national or WHO guidelines [6, 7, 8, 9, 10].

Rwanda, a small East African nation with a population of nearly 13 million, became one of the first SSA countries to implement Treat All nationally in 2016. To date, few data on availability of routine viral load testing in Rwanda have been published, and none after Treat All implementation. Using routinely collected data from ten health centres, we aimed to describe prevalence of viral load testing and viral suppression over a 12‐month interval after a period of ART expansion and viral load scale‐up in Rwanda.

## 2 METHODS

### 2.1 Study design

We conducted a cross‐sectional analysis of routine clinical data collected from January to December 2018 from an open observational cohort of patients receiving HIV care at ten Rwandan health centres affiliated with the Central Africa International epidemiology Databases to Evaluate AIDS (CA‐IeDEA; www.ca‐iedea.org). All research was performed according to the principles of the Helsinki Declaration and was approved by the Albert Einstein College of Medicine Institutional Review Board and the Rwanda National Ethics Committee, both of which waived patient consent because data were de‐identified prior to extraction into the research database.

### 2.2 Setting, clinical procedure and study population

Rwanda implemented Treat All in July 2016, with HIV treatment guidelines stipulating that all newly diagnosed patients should initiate ART as soon as possible after diagnosis [15]. Patients already in care prior to July 2016 but not yet on ART were to initiate ART as soon as possible after Treat All implementation. Under current guidelines, newly diagnosed PLWH (as well as patients who are pregnant or breastfeeding, with concurrent substance use or mental health diagnoses, on third‐line ART, or who are not virally suppressed) are categorized as “unstable” and are scheduled for clinical appointments every three months and pick up medications from the health centre pharmacy every month. A viral load is drawn six months after initiating ART, and if suppressed, yearly thereafter. Patients categorized as “stable” (on ART for ≥18 months with two consecutive suppressed viral loads) decrease the frequency of clinical appointments to every six months, with pharmacy pick‐ups every three months.

For this analysis we included all PLWH ≥15 years receiving HIV care at any of the 10 health centres who: (1) were enrolled in care as of 1 January 2018; (2) had ≥1 health centre visit after enrolment (and could therefore be considered to have engaged in care); (2) had ≥1 health centre visit during 2018; and (3) were not known to have died or transferred out during 2018. Visits included clinical appointments, pharmacy pick‐ups, or laboratory encounters. Each participating health centre routinely collects demographic, clinical and laboratory data as part of clinical care using standardized paper forms; these data are regularly entered into electronic databases and periodically extracted into the CA‐IeDEA research database after de‐identification. Data for this analysis were extracted into the study database on 25 September 2019.

### 2.3 Outcomes and other variables

We defined the following outcomes: on ART (defined as having an available ART initiation date in the study database, with no ART discontinuation prior to 1 January 2018), retained on ART (≥2 post‐ART visits at the health centre in 2018 ≥90 days apart), available viral load test results (viral load measured in 2018 and available in study database) and virally suppressed (most recent 2018 viral load <200 copies/mL, per national guidelines [11]). Demographic and clinical variables included sex (male or female), entry point into HIV care (routine HIV care and treatment, prevention of mother to child transmission (PMTCT), tuberculosis programme), period of enrolment into care (2000 to 2010, 2011 to 2013, 2014 to June 2016 and July 2016 to 2017, corresponding to successive changes in ART eligibility criteria in Rwanda), pre‐ART CD4 count (<200, 200 to 349, 350 to 500 and >500 cells/µL), age in 2018, and the most recently measured body mass index (<18.5 vs. ≥18.5 kg/m^2^) and WHO clinical stage (I or II, III or IV, or missing).

### 2.4 Analyses

We calculated proportions of patients on ART, retained on ART (among those on ART), who had available viral load test results (among those retained on ART), and who were virally suppressed (among those with available viral load results). We then examined factors associated with viral suppression using modified Poisson regression models with robust variances to calculate crude and adjusted prevalence ratios (aPRs) and confidence intervals (CIs), with generalized estimating equations to account for clustering within health centres. Multivariable models were adjusted for all patient characteristics. Data were analysed using SAS 9.4 (SAS Institute Inc., Cary, NC, USA); statistical significance for all tests was two‐sided at *p* < 0.05.

## 3 RESULTS

**Figure 1:**
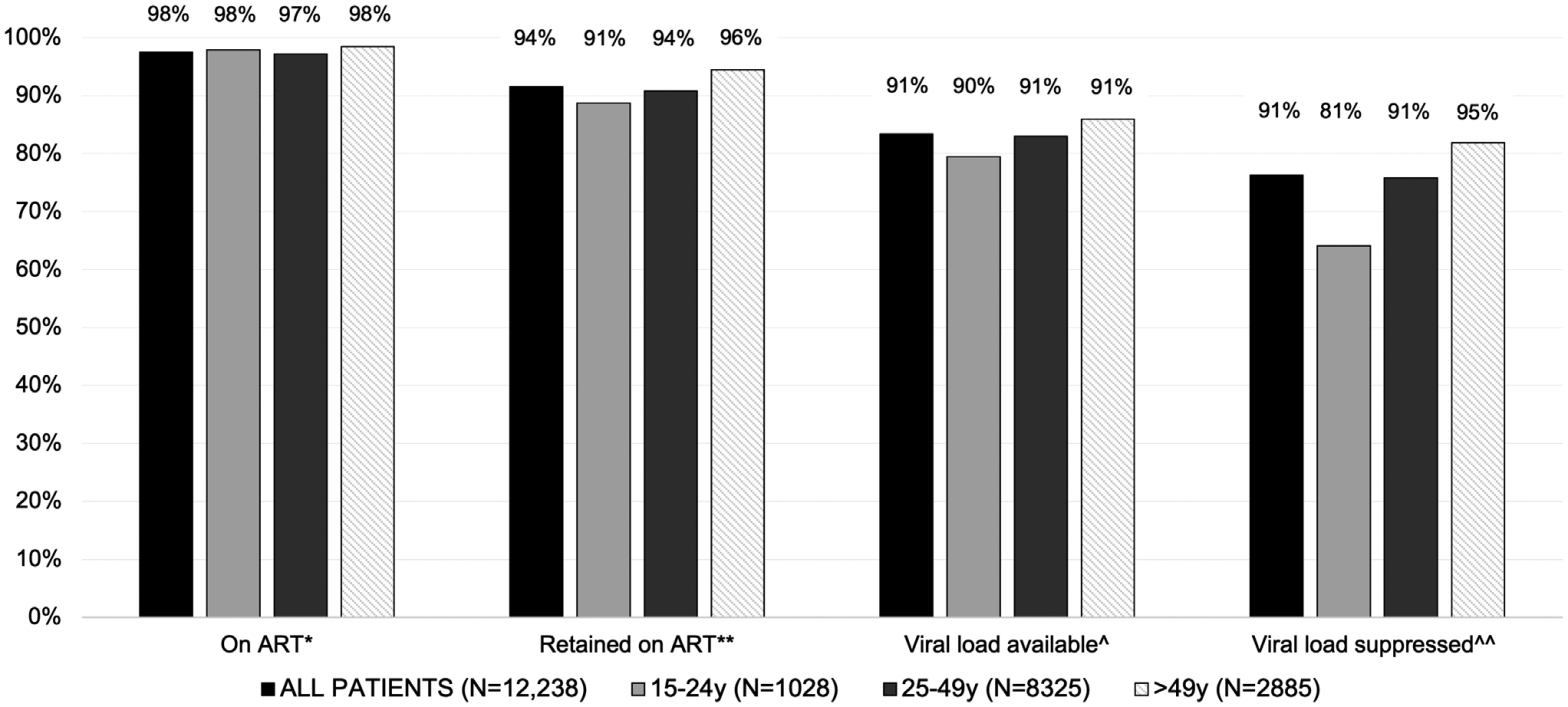
Proportions of patients in HIV care on ART, retained on ART, with available viral load result, and with suppressed viral load, by age – 10 health centres, Rwanda, 2018 (N = 12,328). Note: Percentages above bars indicate proportion of patients meeting outcome among those achieving previous step in cascade. *Available ART initiation date in the study database, with no ART discontinuation prior to 1 January 2018. **≥2 post‐ART health centre visits ≥90 days apart during 2018. ^Viral load measured in 2018 and available in study database. ^^Most recent 2018 viral load <200 copies/mL.

Of 13,287 patients in HIV care in 2017, 1049 (7.9%) did not return for a visit in 2018 and were excluded from further analysis. Among the 12,238 patients in HIV care during 2018, 7050 (58%) were female and 1028 (8%) were aged 15 to 24 years. Most patients (97%) entered directly into routine HIV care and treatment programmes; 248 (2%) and 115 (1%) initially entered care into PMTCT or tuberculosis clinics respectively. Of all patients, 1596 (13%) entered care after implementation of Treat All in July 2016. Median pre‐ART CD4 count was 358 cells/mm^3^ (interquartile range: 240 to 543). In total, 11,933 patients (97%) were on ART, of whom 11,198 (94%) were retained on ART and 10,200 (91% of those retained on ART) had available viral load results (Figure 1). Availability of viral load results in the 10 study health centres ranged from 79% to 96% of patients retained on ART. Patients enrolling in 2014 and after, as well as those with CD4 count <500 cells/mm^3^, were slightly less likely to have an available viral load result (Table S1).

Of the 10,200 patients with available viral load data, 9331 (91%) were virally suppressed. Viral suppression rates in the 10 study health centres ranged from 88% to 94% among patients with available results (Table S2). Viral suppression was less likely among patients aged 15 to 24 and 25 to 49 compared to >49 years (aPRs: 0.83, 95% CI 0.76 to 0.90 and 0.96, 95% CI 0.95 to 0.98 respectively) and those with pre‐ART CD4 counts of <200, 200 to 349 and 350 to 500 cells/mm^3^ compared to ≥500 (aPRs: 0.92, 95% CI 0.90 to 0.93; 0.97, 95% CI 0.96 to 0.98 and 0.97, 95% CI 0.96 to 0.98 respectively; Table 1).

**Table 1:** Prevalence ratio of viral suppression^a^ among patients retained on ART and with viral load measured – 10 health centres, Rwanda 2018 (N = 10,200)

	Total N	Virally suppressed, N (%)	PR (95% CI)	aPR (95% CI)
Enrolment period				
2000 to 2010 (referent)	4826	4480 (93)	–	–
2011 to 2013	2479	2279 (92)	0.99 (0.97 to 1.01)	0.99 (0.97 to 1.01)
2014 to June 2016	1721	1536 (89)	0.97 (0.95 to 0.98)*	0.97 (0.95 to 0.99)*
July 2016 to 2017 (Treat All period)	1174	1036 (88)	0.95 (0.93 to 0.98)*	0.98 (0.94 to 1.03)
Entry point into HIV care				
Routine HIV care and treatment programme (referent)	9837	8996 (91)	–	–
PMTCT	248	228 (92)	1.01 (0.97 to 1.05)	1.00 (0.95 to 1.05)
TB programme	115	107 (93)	1.02 (0.99 to 1.06)	1.03 (1.00 to 1.05)
Sex				
Male (referent)	4289	3898 (91)	–	–
Female	5911	5433 (92)	1.01 (1.00 to 1.03)	1.01 (0.99 to 1.02)
Age in years (2018)				
>49 (referent)	2478	2362 (95)	–	–
25 to 49	6905	6310 (91)	0.96 (0.94 to 0.98)*	0.96 (0.95 to 0.98)*
15 to 24	817	659 (81)	0.84 (0.79 to 0.89)*	0.83 (0.76 to 0.90)*
Most recent BMI (kg/m^2^)				
≥18.5 (referent)	6835	6307 (92)	–	–
<18.5	820	738 (90)	0.98 (0.95 to 0.99)*	0.99 (0.91 to 1.01)
Missing	1000	918 (92)	1.01 (0.96 to 1.06)	1.00 (0.97 to 1.02)
Most recent WHO stage				
I or II (referent)	8017	7358 (92)	–	–
III or IV	1840	1663 (90)	0.98 (0.97 to 1.00)	0.99 (0.97 to 1.00)
Missing	343	310 (90)	0.99 (0.95 to 1.03)	0.98 (0.91 to 1.05)
Pre‐ART CD4 count (cells/mm^3^)				
>500 (referent)	2012	1889 (94)	–	–
350 to 500	1509	1382 (92)	0.98 (0.96 to 0.99)*	0.97 (0.95 to 0.99)*
200 to 349	2067	1900 (92)	0.98 (0.97 to 0.99)*	0.97 (0.96 to 0.98)*
<200	1245	1076 (86)	0.92 (0.91 to 0.93)*	0.92 (0.90 to 0.93)*
Missing	2383	2198 (92)	0.94 (0.92 to 0.96)*	0.95 (0.94 to 0.97)*

ART, antiretroviral therapy; BMI, body mass index; PMTCT, prevention of mother‐to‐child transmission; PR, prevalence ratio; TB, tuberculosis; WHO, World Health Organization.

^a^ < 200 copies/mL on last measured viral load during 2018.

* *p* < 0.05.

Among 1596 patients who entered care after implementation of Treat All, 1484 (93%) were on ART and 1323 of these (89%) were retained on ART. Of those retained on ART, 1174 (89%) had available 2018 viral load results, of whom 1036 (88%) were virally suppressed. In adjusted analyses, there was no statistically significant difference in viral suppression among patients who entered after Treat All implementation compared to those who enrolled before 2010 (aPR 0.98, 95% CI 0.94 to 1.03).

## 4 DISCUSSION

In this study of routinely collected data from HIV programmes in 10 Rwandan health centres during 2018, we found that very high proportions of active patients were on ART, had routine viral load testing performed, and were virally suppressed. These results provide early evidence of the successful scale‐up of both ART and viral load monitoring in a routine clinical setting after Treat All, suggesting that the UNAIDS 90‐90‐90 targets are attainable.

We found high levels of viral suppression among patients whose viral load was measured. This is similar to data published from large controlled trials of universal test and treat in SSA [12, 13, 14] and better than the few observational studies under Treat All published to date from this region [15, 16, 17, 18]. Population‐based HIV impact assessment (PHIA) surveys conducted in several other SSA African countries indicate that similarly high levels of viral suppression are being achieved among patients on ART [19, 20, 21], suggesting that such outcomes are attainable in diverse settings. Our results add to these findings by demonstrating that routine viral load testing can be effectively scaled‐up, providing robust monitoring of ART outcomes at the local and national level.

We found that viral suppression was less likely among patients aged 15 to 24 compared to older patients, results similar to those from multiple other studies in SSA Africa – including HTPN 071 [12] and other large investigations conducted after Treat All implementation [13, 21] – and mirrors preliminary data from Rwanda’s recent PHIA [22]. This study adds to this literature by describing similar outcomes in a setting where nearly all patients were on ART and >85% of them had an available routine viral load. Our results indicate that even with universal treatment, young PLWH continue to lag behind and should be a focus of ongoing efforts to reach the true potential of Treat All. Moreover, our findings suggest that medication adherence may be a driver of lower viral suppression in this age group as minimal differences were observed in receipt of ART or retention on ART when examined by age (data not shown). Implementing evidence‐based approaches to improving ART outcomes for adolescents and young adults will be necessary to address the gap in viral suppression. One potential approach would be building capacity for delivery of adolescent‐focused HIV services, which have been shown to improve care retention and ART adherence in similar settings [24, 25], but are currently available in only 32% of healthcare facilities providing ART in Rwanda [26].

The few studies describing rates of routine viral load testing in SSA indicate that coverage is variable [6, 7, 8, 9, 10]. While some countries have achieved high levels of routine testing [6, 27, 28], in those settings with lower monitoring coverage, estimates of viral suppression are derived from only the proportion of patients whose results are available, potentially over‐ or underestimating the true proportion of PLWH who are virally suppressed. In this study, we observed very high rates of routine viral load testing, though rates were lower in some health centres. However, even in sites where viral load monitoring was less robust, the proportion of those suppressed among those who were tested was consistently high. These results are in agreement with recent research from South Africa suggesting that even in settings where viral load reporting is sub‐optimal, viral suppression estimates may be reflective of the broader population of PLWH on ART [29].

Like any investigation, this study was limited by certain factors. Our use of routine clinical data from patients engaged in HIV care did not allow us to estimate the proportion of PLWH with known HIV status, nor the proportion of those on ART among all PLWH with known status. Because the study focused on viral load testing and suppression, we limited the study to persons who were eligible for testing in 2018 (i.e. those in care in 2018), and thus did not account for patients who may have been lost to care earlier. We report the proportion of those virally suppressed among those with available viral load results, rather than among all those on ART, which could potentially have overestimated the rate of viral suppression. However, the minimal differences between study patients with and without available viral loads, as well similar rates of viral suppression observed in Rwanda’s PHIA [23] suggest that our estimate is fairly accurate. The limited number of available variables did not allow us to measure whether other factors – including comorbid medical or psychiatric conditions, income or education – were associated with viral suppression. Finally, in Rwanda CA‐IeDEA collects data from 10 health centres located in an urban area of a country with a highly functional HIV care service delivery system and with a lower HIV prevalence than in much of SSA, which may limit the generalizability of our findings. However, given the widespread implementation of Treat All, high levels of viral suppression observed in multiple population assessments, and ongoing expansion of viral load monitoring, similar outcomes may be expected in other regions in SSA.

## 5 CONCLUSIONS

In conclusion, we observed high rates of ART use, viral load monitoring and viral suppression among >12,000 PLWH actively engaged in HIV care at 10 Rwandan health centres. These are among the first published routine clinical HIV data after Treat All implementation in SSA and suggest that this policy can effectively contribute to attaining global HIV viral suppression targets.

## COMPETING INTERESTS

All authors have no conflicts of interest to disclose.

## AUTHORS’ CONTRIBUTIONS

JR, DH, QS and KA contributed to the study design, data analysis and interpretation. GM, MY and DN contributed to the study design and interpretation. AM, MR, ER, SN participated in data collection and interpretation. JR drafted the first version of the manuscript, which all authors subsequently reviewed, edited and approved.

## Supporting information


**Table S1.** Characteristics of patients retained on antiretroviral therapy, by availability of viral load – 10 health centers, Rwanda 2018 (N = 11,198)
**Table S2.** Proportion of patients virally suppressed among those with available viral load, by site – 10 health centers, Rwanda 2018
