# Supplementary material for: High levels of viral load monitoring and viral suppression under Treat All in Rwanda – a cross‐sectional study
Source: J Int AIDS Soc. 2020 Jun 14;23(6):e25543. doi: 10.1002/jia2.25543 (PMC7293767; doi:10.1002/jia2.25543)
Supplement: Supplementary file 1 — Table S1. Characteristics of patients retained on antiretroviral therapy, by availability of viral load – 10 health centers, Rwanda 2018 (N = 11,198) Table S2. Proportion of patients virally suppressed among those with available viral load, by site – 10 health centers, Rwanda 2018 [file JIA2-23-e25543-s001.docx]

**Supplemental Table 1.** Characteristics of patients retained on ART, by availability of viral load– 10 health centers, Rwanda 2018 (N=11,198)

|  | **Viral load available (N=10,200)** | **Viral load not available**  **(N=998)** | **p value** |
| --- | --- | --- | --- |
| **Enrollment period** |  |  | <0.001 |
| 2000–2010 | 4826 (92) | 401 (8) |  |
| 2011–2013 | 2479 (91) | 234 (9) |  |
| 2014–June 2016 | 1721 (89) | 214 (11) |  |
| July 2016–2017 (Treat All period) | 1174 (89) | 149 (11) |  |
| **Entry point into HIV care** |  |  | 0.76 |
| Routine HIV care and treatment program | 9837 (91) | 967 (9) |  |
| PMTCT | 248 (92) | 21 (8) |  |
| TB program | 115 (92) | 10 (8) |  |
| **Sex** |  |  | 0.45 |
| Male | 4289 (91) | 432 (9) |  |
| Female | 5911 (91) | 566 (9) |  |
| **Age in years (2018)** |  |  | 0.21 |
| >49 | 2478 (91) | 247 (9) |  |
| 25-49 | 6905 (91) | 656 (9) |  |
| 15-24 | 817 (90) | 95 (10) |  |
| **Most recent BMI (kg/m2)** |  |  | 0.51 |
| ≥18.5 | 6835 (91) | 638 (9) |  |
| <18.5 | 820 (92) | 74 (8) |  |
| Missing | 1000 (93) | 81 (7) |  |
| **Most recent WHO stage** |  |  | 0.14 |
| I or II | 8017 (91) | 802 (9) |  |
| III or IV | 1840 (92) | 157 (8) |  |
| Missing | 343 (90) | 39 (10) |  |
| **Pre-ART CD4 count (cells/mm^3^)** |  |  |  |
| >500 | 2012 (92) | 185 (8) |  |
| 350-500 | 1509 (90) | 162 (10) |  |
| 200-349 | 2067 (91) | 207 (9) |  |
| <200 | 1245 (91) | 127 (9) |  |
| Missing | 2383 (93) | 183 (7) |  |

**Supplemental Table 2**. Proportion of patients virally suppressed among those with available viral load, by site – 10 health centers, Rwanda 2018

| Health Center | Available viral load result,  N | Suppressed viral load,  n (%) |
| --- | --- | --- |
| A | 1842 | 1668 (91) |
| B | 1744 | 1645 (94) |
| C | 2050 | 1899 (93) |
| D | 738 | 660 (89) |
| E | 471 | 417 (89) |
| F | 533 | 486 (91) |
| G | 269 | 238 (88) |
| H | 641 | 593 (93) |
| I | 1481 | 1334 (90) |
| J | 431 | 391 (91) |
